# Association between adjuvant regional radiotherapy and cognitive function in breast cancer patients treated with conservation therapy

**DOI:** 10.1002/cam4.174

**Published:** 2014-04-23

**Authors:** Osamu Shibayama, Kazuhiro Yoshiuchi, Masatoshi Inagaki, Yutaka Matsuoka, Eisho Yoshikawa, Yuriko Sugawara, Tatsuo Akechi, Noriaki Wada, Shigeru Imoto, Koji Murakami, Asao Ogawa, Akira Akabayashi, Yosuke Uchitomi

**Affiliations:** 1 Department of Stress Sciences and Psychosomatic Medicine, Graduate School of Medicine, The University of TokyoTokyo, Japan; 2 Department of Neuropsychiatry, Okayama University HospitalOkayama, Japan; 3 Department of Clinical Epidemiology, Translational Medical Center, National Center of Neurology and PsychiatryTokyo, Japan; 4 Department of Neuropsychiatry, Toshiba General HospitalTokyo, Japan; 5 NISSAN Motor Health Insurance SocietyKanagawa, Japan; 6 Department of Psychiatry and Cognitive-Behavior Medicine, Nagoya City University Graduate School of Medical SciencesAichi, Japan; 7 Department of Breast Surgery, National Cancer Center Hospital EastChiba, Japan; 8 Department of Breast Surgery, Kyorin University HospitalTokyo, Japan; 9 Department of Diagnostic Radiology, School of Medicine, Keio UniversityTokyo, Japan; 10 Psycho-Oncology Division, Research Center for Innovative Oncology, National Cancer Center Hospital EastChiba, Japan; 11 Department of Neuropsychiatry, Okayama University Graduate School of Medicine, Dentistry and Pharmaceutical SciencesOkayama, Japan

**Keywords:** Breast cancer, cognitive impairment, interleukin-6, radiotherapy, Wechsler Memory Scale-Revised

## Abstract

Although protracted cognitive impairment has been reported to occur after radiotherapy even when such therapy is not directed to brain areas, the mechanism remains unclear. This study investigated whether breast cancer patients exposed to local radiotherapy showed lower cognitive function mediated by higher plasma interleukin (IL)-6 levels than those unexposed. We performed the Wechsler Memory Scale-Revised (WMS-R) and measured plasma IL-6 levels for 105 breast cancer surgical patients within 1 year after the initial therapy. The group differences in each of the indices of WMS-R were investigated between cancer patients exposed to adjuvant regional radiotherapy (*n* = 51) and those unexposed (*n* = 54) using analysis of covariance. We further investigated a mediation effect by plasma IL-6 levels on the relationship between radiotherapy and the indices of WMS-R using the bootstrapping method. The radiotherapy group showed significantly lower Immediate Verbal Memory Index and Delayed Recall Index (*P* = 0.001, *P* = 0.008, respectively). Radiotherapy exerted an indirect effect on the lower Delayed Recall Index of WMS-R through elevation of plasma IL-6 levels (bootstrap 95% confidence interval = −2.6626 to −0.0402). This study showed that breast cancer patients exposed to adjuvant regional radiotherapy in conservation therapy might have cognitive impairment even several months after their treatment. The relationship between the therapy and the cognitive impairment could be partially mediated by elevation of plasma IL-6 levels.

## Introduction

As therapies for cancers improve survival time of patients with cancers, protracted cognitive impairment in cancer patients, who do not have tumors in the central nervous system (CNS) and have not had direct therapy to the CNS, has received growing interest in recent years because such impairment often imposes an adverse impact on the quality of life (QOLs) of cancer patients and survivors 1,2.

Recently, cognitive impairment accompanied by radiotherapy not directed to brain areas has been reported. Although Browall et al. found no association between such radiotherapy and cognitive function 3, several studies suggested some association between such radiotherapy and cognitive impairment. While some of these studies suggested that cognitive function recovered during radiotherapy or shortly after radiotherapy 4–6, others suggested that cognitive impairment persisted several months or even several years after radiotherapy 7–12. There were problems with the data interpretation in some of the previous studies. First, many of these studies did not have control groups 3–6,9, or the control groups were not cancer patients 7,10,11. In addition, most previous studies did not perform any objective neuropsychological tests 3–6,8.

With regard to the mechanism of cognitive impairment associated with radiotherapy, several studies suggested that even local radiotherapy induced inflammation and elevated circulating levels of proinflammatory cytokines 13–21. The association of proinflammatory cytokines and cognitive impairment is often referred to in the context of “sickness behavior,” which is a constellation of physiological, behavioral, and neuropsychological symptoms accompanied by conditions which induce inflammation, such as infection and cancer 22,23. In this connection, two clinical studies suggested an association between circulating proinflammatory cytokines and cognitive impairment in cancer patients, and they indicated that only the level of interleukin (IL)-6, among proinflammatory cytokines, including IL-1 and tumor necrosis factor-*α*, had a negative correlation with either cognitive function 24 or cognitive functioning QOL 25, while other proinflammatory cytokine levels had no correlation with it 24,25. Therefore, the elevation of circulating IL-6 levels may be one of the factors important in cognitive impairment in cancer patients treated with radiotherapy.

Accordingly, we hypothesized that one of the mechanisms of cognitive impairment accompanied by radiotherapy not directed to brain areas was that irradiation induces inflammation and elevates circulating levels of proinflammatory cytokines, and among these cytokines, IL-6 plays an important role and leads to cognitive impairment.

The aims of this study were to evaluate whether among non-CNS cancer patients, patients who had undergone local radiotherapy to areas other than brain showed lower cognitive function as assessed by objective neuropsychological tests than patients who had not undergone radiotherapy, and whether elevation of plasma IL-6 levels mediated the cognitive function decline in those patients receiving radiotherapy.

## Material and Methods

This study was approved by the Institutional Review Board and the Ethics Committee of the National Cancer Center of Japan and was performed after obtaining written informed consent from patients.

This study was conducted as a secondary analysis using a database of brain magnetic resonance imaging (MRI) scans from breast cancer survivors 26.

### Subjects and procedures

**Figure 1:**
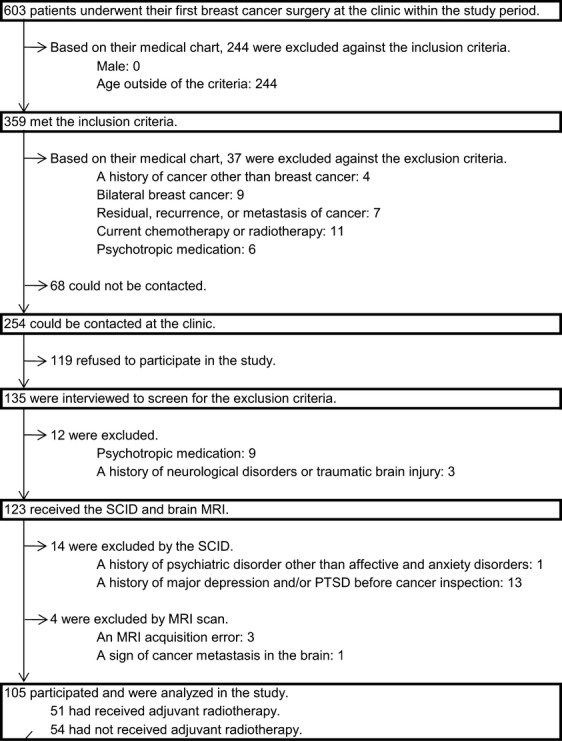
This flowchart illustrates subject sampling in this study. SCID, the Structured Clinical Interview for the Diagnostic and Statistical Manual of Mental Disorders (DSM)-IV; MRI, magnetic resonance imaging; PTSD, posttraumatic stress disorder.

Subjects were recruited during follow-up visits to the Department of Breast Surgery, National Cancer Center Hospital East, after their first breast cancer surgery at the same division. We analyzed their medical charts in continuous sampling and asked the patients who met the inclusion criteria to participate in the study within 3–15 months after their surgery and 1 year after the end of their initial therapy. The patients chosen were (1) women and (2) aged between 18 and 55 years, and did not conflict with the exclusion criteria of (1) a history of cancer other than breast cancer, (2) bilateral breast cancer, (3) clear evidence of residual, recurrent, or metastatic cancer, (4) current chemotherapy or radiotherapy, (5) a history of any neurological disorders, traumatic brain injury, or psychiatric disorders other than affective and anxiety disorders, (6) psychotropic medication taken within 1 month before participation in the study, (7) a history of substance abuse or dependence, (8) a family history of early dementia, (9) any physical symptoms that interfered with daily life, (10) possible dementia defined as a score of <24 on the Mini-Mental State Examination 27,28, (11) a history of major depression and/or posttraumatic stress disorder before inspection for cancer diagnosis, and (12) any contraindication to undergoing an MRI scan. The surgeries were performed from March 1998 to August 2001. Among them, the patients who could be contacted and agreed to participate in the study were interviewed to screen for the exclusion criteria, and the patients who were not excluded received neuropsychological tests, blood sampling, the Structured Clinical Interview for the Diagnostic and Statistical Manual of Mental Disorders (DSM)-IV (SCID) 29, and a brain MRI. The subjects who were not excluded by the exclusion criteria on the SCID and by the MRI data were analyzed (Fig. 1) 26.

The reason why the age for the inclusion criteria was 55 years or under is as follows: a meta-analysis indicated that the prevalence of dementia increases sharply after the age of 65 years 30, and a 14-year follow-up study indicated that the first decline in cognitive performance appears as early as about 10 years before dementia 31. Therefore, in order to exclude the variance of cognitive function associated with dementia as much as possible, we decided that the age of the subjects for this study was 55 years or under.

### Adjuvant regional radiotherapy in breast conservation therapy

Radiotherapy was performed on the remaining breast after breast conservation therapy in the Department of Radiation Oncology, National Cancer Center Hospital East. The method of irradiation for breast conservation therapy followed the clinical practice guideline of breast cancer published by the Japanese Breast Cancer Society 32: 50 Gy tangential irradiation given in 25 treatments to the remaining breast tissue was performed with a radiation source 6 MV X-ray, and in the cases where the resection margin was 5 mm or under from the tumor histopathology, a boost of 10 Gy irradiation was given in five treatments to the tumor bed with a radiation source 6 MeV electron beam.

### Neuropsychological tests

The Japanese version of the Wechsler Memory Scale-Revised (WMS-R) 33,34 was performed. WMS-R consists of indices of Attention/Concentration, Immediate Verbal Memory, Immediate Visual Memory, and Delayed Recall to evaluate memory function 35.

### Plasma IL-6 levels

Blood samples were collected from a peripheral vein into ethylenediaminetetraacetate-2Na tubes and immediately centrifuged at 4°C and 2300*g* for 10 min, and the plasma components were separated and stored at −80°C until analyses. Plasma IL-6 levels were analyzed by automated chemiluminescent enzyme immunoassay (Lumipulse-F, Fujirebio Corporation, Tokyo, Japan). Coefficients of variation in measurements were 2.2–3.8%, and the coefficient of correlation with measurements by traditional enzyme-linked immunosorbent assay by the same company was 0.99 or above 36.

### Statistical analysis

All analyses were performed using SPSS, version 19 (SPSS Inc., Chicago, IL). *α* levels were all set at *P* < 0.05 (two-tailed).

The group differences in each of the demographic or medical factors were compared between the cancer patients exposed to radiotherapy and those not exposed, by using either the Student *t* test, Mann–Whitney *U* test, *χ*^2^ test or the Fisher's exact test.

The group differences in each of the indices of WMS-R were compared between the cancer patients exposed to radiotherapy and those not exposed, using analysis of covariance (ANCOVA) controlling for age, education, accumulated alcohol consumption, smoking status, and body mass index (BMI), which were reported to be associated with impaired cognitive performance 37.

**Figure 2:**
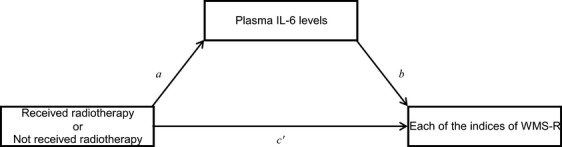
Illustration of a mediation model 38, which hypothesizes that radiotherapy exerts an indirect effect on each of the indices of the Wechsler Memory Scale-Revised (WMS-R) through plasma interleukin (IL)-6 levels. Path *a* represents the effect of radiotherapy on plasma IL-6 levels, the proposed mediator. Path *b* represents the effect of plasma IL-6 levels on each of the indices of WMS-R partialling out the effect of radiotherapy. Path *c'* is the direct effect of radiotherapy on each of the indices of WMS-R partialling out the effect of plasma IL-6 levels. The indirect effect of radiotherapy on each of the indices of WMS-R through plasma IL-6 levels is the product of *a* and *b*, which is tested with the bootstrap confidence interval (CI) obtained through the bootstrapping method.

In order to investigate a mediation effect by plasma IL-6 levels on the relationship between radiotherapy and the indices of WMS-R, the sizes of the indirect effects of receiving radiotherapy on the indices of WMS-R through plasma IL-6 levels were estimated, using a bias-corrected bootstrapping method 38 with 5000 replications, and bootstrap 95% confidence intervals (CIs) were obtained. The outcome variable was each of the indices of WMS-R, the independent variable was whether the patient was exposed to radiotherapy or not, and the mediator was the plasma IL-6 levels. We further controlled for age, education, accumulated alcohol consumption, smoking status, and BMI (see Fig. 2).

In this study, because clinical stage, surgical type, and lymphadectomy had strong correlations with radiotherapy, they were excluded from nuisance values because of multicollinearity (see Table 1).

**Table 1:** Demographic or medical background information in the group of patients exposed to their radiotherapy and in the group of patients unexposed

	Received radiotherapy (*n* = 51)	Not received radiotherapy (*n* = 54)	*P*
Age, mean ± SD, year	47.0 ± 5.2	46.6 ± 6.2	0.755
Handedness: right-handedness, no. (%)	49 (96.1)	53 (98.1)	0.611
Hight, mean ± SD, cm	156.9 ± 6.5	156.0 ± 5.2	0.432
Weight, mean ± SD, kg	56.9 ± 9.0	54.9 ± 6.3	0.196
BMI, mean ± SD, kg/m^2^	23.1 ± 3.4	22.5 ± 2.4	0.333
Education, mean ± SD, year	13.1 ± 1.9	13.2 ± 1.8	0.797
Smoking, no. (%)	8 (15.7)	3 (5.6)	0.116
Accumulated alcohol consumption, mean ± SD, kg	38.4 ± 60.4	27.9 ± 84.6	0.043†
Postmenopausal, no. (%)	29 (56.9)	31 (57.4)	1.000
PS: 0, no. (%)	35 (71.4)^1^	38 (70.4)	1.000
Clinical stage: 0–I, no. (%)	25 (49.0)	13 (24.1)	0.014†
Lymphnode metastasis: positive, no. (%)	15 (29.4)	18 (33.3)	0.824
Histological type, no. (%)			
Carcinoma in situ	4 (7.8)	2 (3.7)	0.428
Invasive carcinoma	39 (76.5)	44 (81.5)	0.696
Special type	8 (15.7)	8 (14.8)	1.000
Histological grade: poor, no. (%)	14 (27.5)	14 (25.9)	1.000
Surgical type: partial mastectomy, no. (%)	51 (100.0)	6 (11.1)	0.000†††
Axillary lymphadectomy, no. (%)	26 (51.0)	44 (81.5)	0.002††
Days after surgery, mean ± SD, day	304 ± 101	270 ± 105	0.102
Radiotherapy: boost irradiation, no. (%)	20 (39.2)	NA	NA
Days after radiotherapy, mean ± SD, day	226 ± 100	NA	NA
Chemotherapy, no. (%)	25 (49.0)	26 (48.1)	1.000
Hormonal therapy, no. (%)	17 (33.3)	15 (27.8)	0.685

NA, not applicable; BMI, body mass index; PS, performance status.

1 Two missing values were excluded.

† Significant difference (*P* < 0.05) between radiotherapy group and no-radiotherapy group.

†† Significant difference (*P* < 0.01) between radiotherapy group and no-radiotherapy group.

††† Significant difference (*P* < 0.001) between radiotherapy group and no-radiotherapy group.

## Results

### Demographic or medical background

Table 1 shows the demographic and medical background data of each group. The subjects consisted of 51 exposed to adjuvant radiotherapy and 54 no-radiotherapy patients (Fig. 1). Because the patients who were exposed to radiotherapy had all chosen breast conservation therapy, their clinical stage was significantly less advanced, and the proportion of patients who underwent axillary lymphadectomy was significantly smaller than that in the no-radiotherapy group. In addition, accumulated alcohol consumption was significantly greater in the group exposed to radiotherapy.

### Radiotherapy and WMS-R

When the difference in each of the indices of WMS-R was compared between the radiotherapy group and the no-radiotherapy group controlling for age, education, accumulated alcohol consumption, smoking status, and BMI, the radiotherapy group showed a significantly lower Immediate Verbal Memory Index and a Delayed Recall Index (radiotherapy group vs. the no-radiotherapy group: 94.9 ± 12.4 vs. 103.6 ± 13.9, *P* = 0.001; 98.5 ± 10.6 vs. 104.3 ± 11.4, *P* = 0.008, respectively. Table 2).

**Table 2:** Each of the indices of WMS-R in the group of patients exposed to their radiotherapy and in the group of patients unexposed

	Received radiotherapy (*n* = 51)	Not received radiotherapy (*n* = 54)	*P*
WMS-R index, mean ± SD			
Attention/concentration	97.4 ± 13.2	101.4 ± 10.3^1^	0.238
Verbal memory	94.9 ± 12.4	103.6 ± 13.9^1^	0.001†††
Visual memory	102.2 ± 9.9	102.4 ± 13.3^2^	0.989
Delayed recall	98.5 ± 10.6	104.3 ± 11.4^3^	0.008††

WMS-R, Wechsler Memory Scale-Revised.

1 One missing value was excluded.

2 Two missing values were excluded.

3 Three missing values were excluded.

†† Significant difference (*P* < 0.01) between radiotherapy group and no-radiotherapy group.

††† Significant difference (*P* < 0.001) between radiotherapy group and no-radiotherapy group.

### Indirect effect of radiotherapy on WMS-R through plasma IL-6 levels

When the size of the indirect effect of receiving radiotherapy on each of the indices of WMS-R through plasma IL-6 levels was estimated controlling for age, education, accumulated alcohol consumption, smoking status, and BMI, the bootstrap 95% CI of Delayed Recall Index only did not include zero (bootstrap 95% CI = −2.6626 to −0.0402), which indicated that the indirect effect was significant (Table 3).

**Table 3:** Regression coefficients between each pair of variables in the mediation models through which indirect effects of receiving radiotherapy on each of the indices of WMS-R through plasma IL-6 levels were estimated (Fig. 2), and bootstrap 95% CIs obtained through the bootstrapping method evaluating these indirect effects

WMS-R index	*a*^1^	*b*^1^	*c'*^1^	Bootstrap 95% CI
Attention/concentration^2^	0.8174†	−1.0133	−1.4550	−3.2207 to 0.1191
Verbal memory^2^	0.8174†	−0.5331	−7.2741††	−2.1231 to 0.3055
Visual memory^3^	0.8173†	−0.3765	1.3768	−1.7209 to 0.3672
Delayed recall^4^	0.8138†	−1.1678	−4.6102†	−2.6626 to −0.0402‡

WMS-R, Wechsler Memory Scale-Revised; IL-6, interleukin-6; CI, confidence interval.

1 Regression coefficient between each pair of variables corresponding with each symbol representing each path in Figure 2.

2 The plasma IL-6 levels and the index of WMS-R of 96 patients (received radiotherapy 49 and not received 47) were available for analysis.

3 The plasma IL-6 levels and the index of WMS-R of 95 patients (received radiotherapy 48 and not received 47) were available for analysis.

4 The plasma IL-6 levels and the index of WMS-R of 94 patients (received radiotherapy 47 and not received 47) were available for analysis.

† *P* < 0.05.

† *P* < 0.01.

‡ The indirect effect mentioned was significant at *α* level *P* < 0.05.

## Discussion

This study showed that breast cancer patients exposed to adjuvant regional radiotherapy in breast conservation therapy at 7 months after treatment showed a significantly lower Immediate Verbal Memory Index and a Delayed Recall Index of WMS-R than breast cancer patients not exposed to radiotherapy. We also found that radiotherapy exerted an indirect effect on the lower Delayed Recall Index of WMS-R through elevation of plasma IL-6 levels. These results suggested that adjuvant regional radiotherapy in breast conservation therapy could impair memory function some months after completion of the therapy, and that the influence of the therapy on the impairment of memory function is partially mediated by elevation of plasma IL-6 levels.

There have been three studies on the relationship between radiotherapy and cognitive function by objective neuropsychological tests in breast cancer patients. One study was cross-sectional showing significantly lower attention and complex cognition in the Trail Making Test in the patient group exposed to radiotherapy than that in the non-cancer control group 7. Another study was longitudinal from before and up to 3 months after radiotherapy and showed a decline from baseline in verbal memory in the Rey Auditory Verbal Learning Test 11. The third study was longitudinal at 6 months and at 36 months after radiotherapy showing a significantly smaller improvement in processing speed and significantly lower executive function on a subtest of the Wechsler Adult Intelligence Scale III at both time points in the patient group exposed to radiotherapy than that in the non-cancer control group 10. The results of this study using WMS-R (Table 2) generally support these findings. However, this study had an advantage over these previous studies because the previous studies did not have a control group consisting of breast cancer patients who had not been exposed to radiotherapy. Therefore, this study provided more compelling evidence that cognitive impairment was caused by radiotherapy, not by cancer itself and/or by treatments other than radiotherapy.

This study suggested that adjuvant regional radiotherapy in breast conservation therapy might elevate plasma IL-6 levels as a byproduct of the analysis of the indirect effect of radiotherapy on the indices of WMS-R through plasma IL-6 levels (Table 3), although the relation between radiotherapy for cancer patients and the levels of circulating proinflammatory cytokines after radiotherapy has been inconsistent in previous studies, that is, some studies showed elevated levels after irradiation 16,20, but others showed the opposite results 4,14,21,39. The mechanism for the elevation of plasma IL-6 levels is not known and should be investigated in future studies. It may be added that the clinical stage was significantly less advanced in the radiotherapy group than that in the no-radiotherapy group in this study (Table 1). Thus, the possibility that advanced clinical stages influenced prolongation of high level of plasma IL-6 after radiotherapy seems to be low in this study.

This study showed that radiotherapy exerted a significant indirect effect through plasma IL-6 levels only on the Delayed Recall Index of WMS-R (Table 3). It has been suggested that delayed recall memory is associated with the hippocampus 40,41. Furthermore, an animal study suggested that peripheral IL-6 signaled the brain and induced inflammation in the hippocampus 42. Therefore, the association between radiotherapy and memory function impairment might be explained partially by hippocampal inflammation caused by the elevation of plasma IL-6 levels, while adjuvant chemotherapy did not influence the hippocampal volume in breast cancer survivors 26,43.

There were some limitations to this study. (1) This study was not an interventional study, and was a cross-sectional study. Therefore, the causality between variables was not guaranteed. (2) Because there was a considerable length in time span between the end of the therapies and the search points, the variance of measurements may be larger than if all searches had been performed at the same time after the therapies ended. This can reduce the power of the tests in this study. (3) The number of subjects was small. Therefore, the power of the tests might be reduced. (4) Because the subjects in this study were restricted to comparatively young breast cancer patients, the results should be generalized with caution. (5) The influence of residual cancer on inflammation could not be excluded. (6) Neuropsychological tests other than WMS-R were not conducted in this study. (7) Some factors other than plasma IL-6 that might be associated with cognitive impairment accompanied by radiotherapy, such as other proinflammatory cytokines, fatigue, anemia, chronic pain, etc., were not considered in this study. (8) Biological factors which might have elevated plasma IL-6 levels, such as medication, infection, etc., were not considered in this study.

## Conclusion

Breast cancer patients exposed to adjuvant regional radiotherapy could have cognitive impairment, which might be partially mediated by the elevation of plasma IL-6 levels.
